# Neurological Complications of COVID-19: A Review of the Literature

**DOI:** 10.7759/cureus.27633

**Published:** 2022-08-03

**Authors:** Lucy Dale

**Affiliations:** 1 Foundation Year Doctor, Ninewells Hospital and Medical School, Dundee, GBR

**Keywords:** public health care, long covid, spike protein and covid-19, intensive care unit stay, neuro-immunology, neuroinflammatory, neurological disease, brain and spine, stroke and covid-19, coronavirus disease 2019 (covid-19)

## Abstract

Coronavirus disease 2019 (COVID-19) has caused the most unprecedented health crisis since the 1918 H1N1 pandemic. Whilst COVID-19 is traditionally considered to be a respiratory disease, it is important to understand that this virus has the potential to disseminate throughout the body causing multi-organ failure. Both peripheral and central neurological systems have been shown to be greatly affected. This review aims to look at the available literature published on COVID-19 and summarize the main neurological complications seen so far.

## Introduction and background

Arguably, COVID-19 has caused the most unprecedented global health crisis since the 1918 H1N1 pandemic. The virus was originally identified in Wuhan, China, in December 2020. Over two years on, it is estimated that severe acute respiratory syndrome coronavirus 2 (SARS-CoV-2) has infected 572 million individuals and caused over 6.3 million deaths worldwide [1]. The magnitude of turmoil the virus has created is so severe that additional hospitals (for example, Nightingale in Central London) have been constructed to support the incredible demands placed upon healthcare systems across the world.

Severe acute respiratory syndrome coronavirus 2 (SARS-CoV-2) is classified to be a part of the Coronaviridae family [2]. There are multiple coronavirus strains, ranging in clinical severity; from the common cold to severe acute respiratory distress syndrome (SARS) and Middle Eastern respiratory distress syndrome (MERS). Genomically, SARS-CoV-2 is a single-stranded, positive-sense, enveloped virus. It possesses a variety of membranous glycoproteins that attribute to its pathogenic nature, for example, the spike (S) glycoprotein [3]. Over the past few months, the S-glycoprotein has created anxiety for the scientific community and public because it has shown the ability to mutate, resulting in highly contagious strains. The clinical significance of the new strains is that there is a reduced threshold for infectivity, thus vulnerable individuals are at a greater risk of contracting the virus and becoming critically unwell.

Whilst it is acknowledged amongst the scientific community and public that SARS-CoV-2 affects the respiratory system, the virus has shown the ability to quickly disseminate throughout the body affecting multiple organs [4].

The nervous system is subdivided into two parts: the central nervous system (CNS) and the peripheral nervous system (PNS) [5]. The CNS consists of the brain and spinal cord. The peripheral nerves reside outside of the brain and spinal cord, that is, cranial nerves I-XII which supply the head/neck, and spinal nerves which supply the motor, sensory, and autonomic function of the rest of the body. This review aims to discuss and explain the neurological complications seen in COVID-19.

At the start of the pandemic, most literature published on the neurological complications of COVID-19 were small case reports/series [6-7]. Over a year on, there is now a plethora of scientific evidence available detailing various neurological complications observed in patients infected with SARS-CoV-2. The virus has been shown to affect both the central and peripheral nervous systems, with a range of clinical impacts [8]. It has been theorized that the pathophysiological mechanisms of COVID-19 on the nervous system are caused by entry into host cells via viral glycoproteins, which then results in a widespread inflammatory response of the immune system and vasculature [9-10]. However, it is appreciated that more research needs to be done to fully understand this.

Aims and methods

The aim of this literature review was to identify the different neurological complications associated with COVID-19 infection. The methods were to find the most relevant, appropriate literature on this subject.

A search strategy was adopted to yield papers using the PubMed database. Specific words were searched in combinations, for example “COVID-19,” “Common Neurological,” “Neuroinflammatory,” “Neurovascular,” and “Neuropsychiatric”. In addition to using results from the database, other resources including website pages were searched.

**Figure 1:**
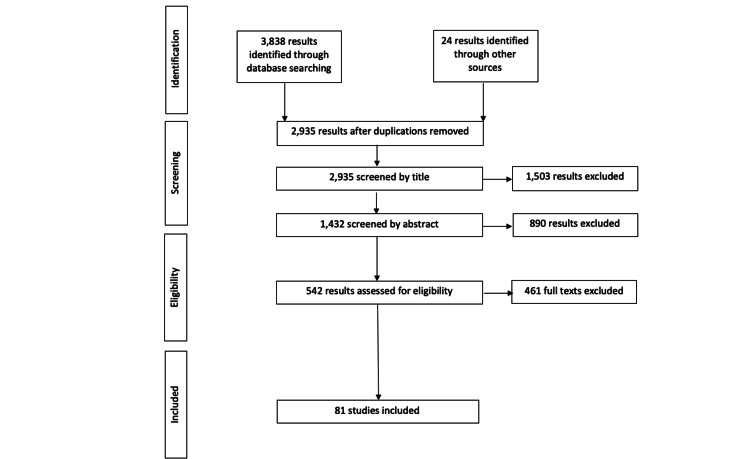
PRISMA flow chart.

For ease of review and relevance, a Preferred Reporting Items for Systematic Reviews and Meta-Analyses (PRISMA) strategy was adopted (Figure 1) [11].

Duplicate results were removed initially, followed by a screening of the literature by title and abstract. The aim of this initial screening process was to identify papers that specifically fit into the question at hand. A large amount of literature yielded during the initial search result was irrelevant (for example, focusing on a different bodily system).

Following this, the literature was assessed for eligibility, with full text being screened. There is a large number of individual case reports/reviews available on specific neurological complications, therefore, one to two articles were chosen on specific topics that were felt to be clearest in conveying results. Overall, there was a good amount of relevant literature; providing the platform to write this review.

This review will initially focus on the most common benign neurological complications of COVID-19, followed by the more serious ones observed. Complications involving the central and peripheral nervous system will be discussed, along with neuropsychiatric effects as they sit on the border between neurological and psychiatric disease. For completeness, some of the rarest and most interesting neurological complications documented will be reviewed.

## Review

Common benign neurological complications

It is arguable that the most common benign neurological-related complications of COVID-19 documented within scientific literature are anosmia and/or ageusia (the loss of smell and/or taste) [7, 12-13]. Whilst it is appreciated that coronaviruses commonly cause a loss of smell and/or taste, these symptoms were originally not promoted to be a headline feature of SARS-CoV-2’s symptomatology. Indeed, it was not until two months after the arrival of the virus into the United Kingdom that the government and National Health Service promoted the importance of these symptoms to the public [14].

The olfactory system is derived from the nervous system [15]. Its embryological origin consists of the olfactory epithelium (PNS) and olfactory bulb (CNS). Pathophysiologically, it is thought that COVID-19’s spike protein interacts with angiotensin-converting enzyme 2 (ACE2), and transaminase protein serine 2 (TMPRSS2) receptors on olfactory neurons [16-17]. This interaction between the S-protein with olfactory receptors creates difficulty for odors to bind to the neuroepithelial cells, causing a loss of smell. The gustatory system is also closely related to the nervous system [18]. Gustatory cells are supplied by cranial nerves VII, IX, and X, and the sense of taste is expressed within the tongue and palate. Again, it is thought that the SARS-CoV-2 spike glycoprotein possesses neuroinvasive properties in that it binds to ACE2 receptors on the oral mucosa. This leads to an inflammatory cascade within the mucosal cells, disrupting the tongue and palate receptors that help relay taste signals to the CNS.

With regard to the prevalence of these two symptoms, a systematic review and meta-analysis published by Agyeman et al. [19] reported that approximately 40% and 38% of patients diagnosed with COVID-19 experienced some degree of alteration to their sense of smell and taste respectively. This statistic highlights how common these symptoms can be within the general population. In saying this, literature has identified an increased prevalence of anosmia/ageusia in younger patients, and the exact reason for this is yet to be understood. Younger patients infected with COVID-19 tend to experience more benign complications, regardless of the bodily system [20]. This contrasts with the elderly cohort who unfortunately tend to become more critically unwell [21].

A large proportion of individuals infected with SARS-CoV-2 experience anosmia/ageusia temporarily, with the average length of time being seven days from symptom onset [22]. Nevertheless, there is documentation within literature indicating that some patients suffer from these symptoms for extended periods -- i.e., weeks/months, with a few experiencing a chronic loss of their sense of taste/smell that has not returned to normal [23-24].

Another neurological symptom of COVID-19 that is often seen as a complication for many, is a headache. The pathophysiological mechanism of the virus to cause headaches is complex. Irritation of the meninges caused by viral proteins, pro-inflammatory cytokines, and hypoxia all contribute to the sensation of headache. Whilst this symptom usually subsides within a week, there is unfortunately a cohort of patients who require ongoing help from primary care physicians to help deal with debilitating headaches (such as migraines) that occur for a long time after initial infection [25].

Even though the most common complications are often thought to be the most benign, they should not be ignored. The literature has highlighted that these symptoms can cause a significant impact on patient quality of life, and thus overall wellbeing.

Neurovascular complications

Whilst most patients infected with COVID-19 do not develop any serious complications requiring medical treatment, there is a proportion that unfortunately does. One of the more prevalent severe neurological complications seen in patients with COVID-19 is cerebrovascular events, resulting in neurological deficits [26-28]. To highlight this, the literature identifies the prevalence of cerebrovascular accidents (CVAs) in patients who are COVID positive to be as high as 6% [29]. This section will look at both arterial and venous CVAs observed in COVID-19 patients.

Arterial CVA’s can be broadly split into ischemic strokes and hemorrhagic strokes [30]. Arterial CVAs most commonly affect the elderly cohort and those with relevant risk factors, i.e., overweight/obesity, hypertension, hyperlipidemia, and diabetes [31]. The available literature reports cases of COVID-19 patients experiencing both types of strokes (ischemic and hemorrhagic). The commencement of anticoagulation therapy has been shown to increase the risk for the development of hemorrhagic events in patients with COVID-19 [32-33]. In contrast, immobility and hypercoagulability seen in COVID-19 patients are thought to contribute to the development of ischemic stroke [34-36]. The development of a hypercoagulable state in COVID-19 patients is due to the widespread inflammatory processes COVID-19 causes within the vasculature, often referred to as a “cytokine storm.” This is where the virus causes the body to produce high levels of inflammatory and pro-coagulative factors, including CRP, D-dimer, IL-6, and fibrinogen. The resultant is often observed later in the disease process, and by this point, patients are already admitted into secondary care [26].

The symptoms of arterial CVAs in patients who are non-sedated/non-intubated present with are identical to non-COVID stroke symptoms. This includes but is not limited to, sudden-onset hemiparesis, hemisensory loss, diplopia/hemianopia, ataxia, and speech problems [36]. Following a detailed history of symptoms and relevant neurological examination, patients receive the appropriate investigations. This includes routine blood tests (e.g., FBC, U&Es, LFTs, d-dimer, CRP), electrocardiogram to check for atrial fibrillation, and appropriate imaging, such as diffusion-weighted MRI brain or CT-head. The results of the investigations are necessary to determine the nature of the CVA, and what treatment pathway to begin.

If a transient ischemic attack (TIA) is suspected, the ABCD2 scoring system can be used which helps physicians determine the risk of developing a stroke following a TIA. The treatment for TIAs is variable, however, most patients are given an antiplatelet medication such as aspirin and should be ideally seen by a stroke physician within 24 h for further management [37]. Regarding the treatment of ischemic strokes, patients should be admitted immediately to a stroke unit for further workup and may receive either medical (thrombolysis) or surgical (thrombectomy) intervention, followed by preventative management including anticoagulation, and blood pressure/cholesterol management [38]. Hemorrhagic strokes are often managed through surgical intervention. For the more severe COVID-19 patients where respiratory function is also compromised, escalation to the ICU is often needed for further monitoring due to the risk of deterioration [27, 39].

For COVID-19 patients who are intubated with an endotracheal tube and/or heavily sedated, it is much more difficult to appreciate when a CVA has occurred [40]. This often means patients go for longer periods undiagnosed. As mentioned earlier, patients who are admitted into hospital, particularly those that go to the ICU, are at a significantly higher risk for the development of a CVA.

Identification of an ischemic event in patients who are intubated/sedated and not paralyzed may be observed through muscular spasticity, or abnormal limb positioning, such as elbow/wrist flexion, with internal rotation and adduction of the shoulder. Post-stroke spasticity can often take a long time to occur and is, therefore, often missed. For hemorrhagic strokes, patient pupils are regularly checked for anisocoria, i.e., unequal pupil size [41-42]. Again, this can often be subtle. Therefore, identification of CVAs in the ICU regardless of etiology is often made when patients are being weaned off sedation, and they respond in a neurologically inappropriate way [43] (e.g., lack of appropriate limb movement). If this occurs, patients receive appropriate brain imaging.

The treatment of CVAs in COVID-19 patients who are intubated is based on many different factors, including overall health status, age, and suspected level of damage. For example, there have been cases where unfortunately very young patients with COVID-19 have suffered from large cerebrovascular events with suspected irreversible damage [29, 42]. 

The points discussed above look at neurological complications that have arisen from problems within the arterial vasculature. COVID-19 can also cause complications within the venous vasculature, again resulting in neurological deficits. Cerebrovascular complications of the venous system are much rarer than arterial. However, one of the more serious venous complications seen in COVID-19 patients is cerebral venous sinus thrombosis (CVST) [44-46]. This is where a thrombus forms within the venous vasculature of the brain, such as the superior sagittal sinus, transverse sinus, sigmoid sinus, and even the jugular foramen. The more severe CVSTs can interrupt the drainage of blood from out of the brain, which poses the risk of hemorrhagic transformation.

In contrast to arterial complications, CVSTs typically affect younger adults [45-46]. Predisposing risk factors for venous thromboses are slightly different from that of arterial. There is less focus on modifiable risk factors, and instead, hypercoagulopathies are considered more relevant. For example, pregnancy and genetic coagulopathies such as sickle cell/beta-thalassemia, place patients at a significantly higher risk. In saying this, the inflammatory and hypercoagulable state of COVID-19 causes puts patients at risk, irrespective of the clot location within the vasculature [44-46].

The symptoms of CVST patients with COVID-19 can experience include headaches, blurred vision, seizures, and loss of consciousness. Regardless of intubation status, one clinical sign that is looked for in patients with suspected CVST, is papilledema. This is because CVST can cause intracranial hypertension [46].

As with suspected arterial accidents, venous accidents are investigated through simple investigations including blood tests, electrocardiogram (ECG), and imaging including magnetic resonance (MR) or CT venogram. Treatment of venous accidents must be rapid and can either be medical (e.g., thrombolysis/anticoagulation/antiepileptics) or surgical (thrombectomy/decompressive craniotomy). Again, the direction of treatment in COVID-19 patients depends on clinical stability [44-46].

Whilst some patients with COVID-19 who experience a CVA make a full recovery, there are many cases, particularly those in the ICU, where CVAs cause devastating long-term neurological complications. A study published by Ntaios et al. [47] found that patients with COVID-19 who experienced a CVA have worse rates of morbidity/mortality, and quality of life, than non-COVID stroke patients. This was reflected through an overall lower modified Rankin scale, which measures the degree of disability post-stroke.

Cerebrovascular complications of COVID-19 are worrying. The literature available details very young patients with no cardiovascular risk factors developing such complications highlighting the virus’s ability to affect anyone of any age, regardless of baseline clinical status.

Neuroinflammatory complications

COVID-19 can cause inflammation within parts of the nervous system. This can be anatomically divided into meningeal (meningitis) and brain parenchyma (encephalitis) inflammation. The latter can progress into the worrying clinical state physicians describe as “encephalopathy.”

The pathogenic mechanisms of COVID-19 in causing meningeal and encephalic inflammation are complex, and it must be said that meningoencephalopathy directly caused by COVID-19 is rare. As discussed in the common complications section of this review, COVID-19 can infect olfactory neuroepithelium. This can result in the retrograde transfer of virion particles into the CNS [48]. Moreover, it is thought that viral particles infect the CNS via the blood-brain barrier during the initial stage of infection. Following the penetration of the virus into the CNS, an intense inflammatory cascade resulting in meningeal/encephalic inflammation and irritation occurs [48-50]. This, coupled with hypoxia, is why patients with COVID-19 can present with the severe neuroinflammatory disease.

COVID patients who possess underlying co-morbidities or those who receive immunosuppression therapy (e.g., steroids) have been shown to increase the risk of opportunistic infections developing within the body, including neuroinflammatory diseases such as bacterial, fungal, or alternative viral meningoencephalitis [51].

Regardless of whether COVID-positive individuals develop a primary or secondary neurological infection, the clinical presentation for both is roughly the same. For pure meningitis, patients can present with fevers, headaches, and neck stiffness [49]. For encephalitis, the presentation is often more severe and can progress to encephalopathy. This is defined as a widespread brain disease with resultant effects on brain structure/function [52]. Symptoms of encephalopathy which highlight the gross injury to the brain include depressed mental status, which can result in a coma. In addition to this, patients can have severe seizures due to interrupted neurological activity within the brain. In reality, both meningitis and encephalitis can occur together resulting in a devastating picture of meningoencephalitis/encephalopathy.

Investigating the neuroinflammatory complications of COVID-19 include routine blood tests, and imaging, including CT-head to identify any raised intracranial pressure (ICP) [48-49, 52]. Patient cerebrospinal fluid (CSF) may also be analyzed via lumbar puncture. The CSF analysis includes looking at the appearance of the fluid and the levels of protein, glucose, and white cell count. To identify potential bacterial infections that are secondary to COVID-19, CSF Gram stain, and microscopy, culture and sensitivity can be sent to the laboratory for further analysis. For suspected primary (COVID-19) or secondary (HSV, etc.) viral neuroinflammatory disease, viral polymerase chain reaction (PCR) can be sent to the lab. There have only been a few cases within the literature identifying patients with a positive COVID-19 PCR seen within the CSF [53-54], again, highlighting the rarity of this diagnosis.

Treatment of meningoencephalitis in COVID-19 patients depends on the causative organism. For secondary superimposed bacterial infections, IV antibiotics including ceftriaxone and vancomycin are used [55]. For meningoencephalitis where the cause is thought to be a primary infection, i.e. COVID-19, treatment is mainly supportive. This includes symptom management, such as anti-epileptics for seizure control. Occasionally, medications including hydroxychloroquine, IV methylprednisolone, and IV immunoglobulins are used for immunosuppressive control. Occasionally, IV acyclovir is also prescribed to empirically cover the risk of HSV encephalitis, even if this is not identified within CSF analysis [56].

It can be argued additional neuroinflammatory complications of COVID-19 occur in individuals who already suffer from neuroinflammatory conditions, mainly multiple sclerosis (MS). MS is a chronic immune-mediated and neurodegenerative disorder, developed by both genetic and environmental factors [57]. Common symptoms of MS include fatigue, visual disturbances, and motor/sensory impairment. Interestingly, studies have shown that patients with MS are at a higher risk of developing neurological symptom recurrence preceding or coinciding with COVID-19 infection. For example, Garjani et al. [58] identified 57% of patients experiencing an exacerbation of MS symptoms during COVID-19 infection. Whilst there is no definitive cure for MS, physicians may offer patients steroids during relapses, or disease modifying therapies (DMTs) to reduce the number of relapses. Regarding COVID-19, the literature is limited in identifying whether DMTs reduce symptom recurrence. 

The overall clinical outcome for COVID-19 patients with neuroinflammatory disease depends on a multitude of factors. Whilst some patients make a full recovery, patients who fall ill may experience long-term neurological problems [59].

Neuropsychiatric complications

It is important to touch on the neuropsychiatric complications of COVID-19, because these complications sit on the border between neurological and psychiatric illness and are, therefore, relevant from a neurological perspective [60].

One of the most common neuropsychiatric complications seen in patients with COVID-19 is delirium [61]. This is often related to prolonged ICU admission and thus indirectly related to COVID-19. The literature suggests COVID-19-related delirium is more prevalent compared to non-COVID-19 delirium [62]. The etiology for this includes factors including neuroinflammation, secondary infections in combination with environmental factors. The ICU is an unfamiliar environment for many, and with the strict personal protective equipment (PPE) measures that have been put in place since the start of the pandemic, many ICU patients go long periods without seeing a human face that is not behind a mask.

The importance of delirium is that literature has associated patients with having both poorer clinical and functional outcomes [60-61]. A study published by Mcloughlin et al. [63] found that COVID- 19 patients who experienced in-hospital delirium had worse functional outcomes post-discharge, that is, they struggled more whilst doing their activities of daily living than those who did not experience delirium throughout their stay. This finding remained statistically significant (p<0.01) even after appropriate patient factor adjustments. It is thought that patients who experience delirium have worse long-term outcomes because the etiology of delirium often corresponds with other illnesses (e.g., secondary infection) that patients experience throughout their battle with COVID-19.

A more severe and rare neuropsychiatric complication of COVID-19 that has been documented is catatonia, and only a few publications have reported this [64-65]. The etiology of COVID-19 catatonia has not yet been extensively explored. Authors have theorized that this rare presentation could be iatrogenic in etiology or be caused by the severe mental stress patients must deal with whilst in the ICU.

Furthermore, a condition that some scientists have argued to have neuropsychiatric elements is “Long COVID” [61]. Some researchers believe Long COVID is like functional neurological illnesses including chronic fatigue syndrome (CFS) and functional neurological disorder (FND) [66]. Neuropsychiatric symptoms patients with Long COVID might experience include depression, anxiety, and cognitive deficits [67]. Data collected from the U.K’s COVID-19 infection survey [68] estimates that around 10% of people who test positive for the virus experience Long COVID, that is, symptoms that last for greater than three months. Therefore, this complication should be discussed as it has a significant impact on many people.

Long COVID is defined as ongoing multi-organ complications and the inability to recover from symptoms for weeks to months following initial infection. The neurological symptoms of Long COVID include extreme tiredness, amnesia, dizziness, and insomnia [66]. Long COVID can have a debilitating impact on the quality of life due to the length of symptoms post-infection [69]. Therefore, this complication is extremely important. 

Overall, the neuropsychiatric complications observed in COVID-19 are wide-ranging and can affect a large proportion of patients significantly.

Peripheral complications

As mentioned in the introduction, COVID-19 can affect the peripheral nervous system.

Important peripheral neurological complications observed in critically unwell COVID patients include critical illness polyneuropathies (CIP) and critical illness myopathies (CIM) [70-71]. CIP and CIM are diseases of the peripheral nerves in patients who have experienced severe trauma and/or critical illness. The literature has identified the prevalence of peripheral neuropathies to be higher in COVID patients than in non-COVID patients. To support this statement, an observational study written by Frithiof et al. [71] reported that 9.9% of COVID-19 patients developed CIN/CIM, whereas only 3.4% in the general population developed CIN/CIM.

The clinical presentations of CIM and CIN are slightly different. CIM affects the nerves supplying motor function, resulting in significant atrophy, with sensory function usually being preserved. Furthermore, CIM generally affects the proximal aspect of the limb. This contrasts with CIP, which usually affects the distal limb, and is characterized by impaired sensory function. Whilst CIP also causes weakness, the level of atrophy is usually less compared to that seen in CIM due to a degree of motor neuron preservation.

Long-term outcomes for patients with critical illness neuromyopathy (CINM) are mixed. It must be said that patient factors including age, previous mobility, and co-morbidities are very important in determining how well this disease resolves. Nevertheless, a large proportion of patients can continue to suffer from chronic neurological deficits like foot drops and sensory changes like paraesthesia or chronic pain for up to months post-discharge [72].

Treatment for CIN/CIM is mainly supportive. Early intense physiotherapy regimes are recommended to aid the restoration of baseline sensory and motor function. For patients whose glucose levels are raised, extensive dietician input and tight glucose control with insulin therapy are suggested. This is necessary because high glucose levels result in oxidative stress and free radical formation, resulting in further damage to peripheral nerves [73-74].

The above conditions primarily affect the upper and lower limbs. What is interesting is that literature has also identified a link between COVID-19 affecting peripheral nerves of the head and neck. The facial nerve (CN VII) carries motor fibers to the muscles of facial expression, parasympathetic fibers to the lacrimal and salivary glands, and special sensory fibers to supply taste to the anterior two-thirds of the tongue [75]. In facial nerve palsy, patients typically present with total unilateral paralysis of the muscles supplied by CNVII, ptosis, and lacrimal/salivary gland disturbances. COVID-19 has been identified by a number of authors to potentially cause facial nerve palsy [76]. This is thought to be due to the viruses' neuroinvasive abilities to directly infiltrate the nerve fibers. 

Another associated peripheral neurological complication of COVID-19 that has been documented is trigeminal nerve (CN V) neuralgia [77]. This is where patients experience sudden, severe facial pain in the forehead, cheek, and lower jaw. However, the evidence for this association is weak. 

The rarer COVID-19 peripheral neurological complications documented within the literature are extremely interesting. A few case studies published in the U.K. have identified Guillain-Barre syndrome (GBS) as a potential post-infectious complication of COVID-19 [78-79]. Even outside of the COVID world, GBS is a rare autoantibody inflammatory peripheral neurological disorder, typically caused by infection with *Campylobacter Jejuni*, a Gram-negative bacterium [80].

Webb et al. [79] first made the association between COVID-19 and GBS in 2020, with documentation of a 57-year-old male presenting to the emergency department with ascending paralysis one week after diagnosis with COVID-19. This eventually progressed into respiratory failure, with intubation being needed (the overall outcome of this patient has not been documented). Whilst GBS is an incredibly rare complication, clinicians should be aware of this, and if suspected, must act quickly without delaying treatment. The treatment for GBS can be with plasmapheresis which helps remove autoantibodies from the blood or with IV immunoglobulin which provides antibody replacement to help stop the autoantibodies produced from causing further nerve damage. Supportive treatment is also provided.

Another interesting peripheral neurological complication of COVID-19 is Miller-Fischer syndrome (MFS) [81-82]. Again, this disease is rare and considered to be a variant of GBS. Cases have been identified where COVID-19 patients present with diplopia due to weakening of the ocular musculature, ataxia, and reduced reflexes. As with GBS, the treatment of MFS is IV immunoglobin. Again, whilst only a few cases of this disease have been documented, it is important for clinicians to remain vigilant and to include this as part of their differential diagnosis.

Rare complications

Whilst it is important for clinicians to have a sound understanding of the more common neurological complications of COVID-19, it is also useful to understand rarer complications that do not directly fit into a category.

There have been some fascinating cases documented over the past year. For example, a UK-wide surveillance survey published by Varatharaj et al. [83] looked at the different neurological complications of COVID-19. It was reported that one patient presented with an opsoclonus- myoclonus syndrome (OPS); an incredibly rare neurological disorder that results in opsoclonus (rapid eye movements, occurring in any direction), myoclonus (fast involuntary muscular jerks), and ataxia. The prevalence of OPS in the general population is thought to be 0.18 cases per million [84]. A small case series analysis conducted by Emamikhah et al. [85] identified opsoclonus-myoclonus syndrome as a post-infectious complication of COVID-19, with the average symptom onset being 11 days. Whilst some clinicians believe that the presentation of OPS whilst infected with COVID-19 are separate issues, there is a school of thought that COVID-19 causes this rarity. Whilst the pathological mechanism of this is complex, it is thought to be immunologically mediated.

Another rare neurological complication seen in COVID-19 patients that might be caused directly by the virus is an isolated sixth nerve palsy [86]. It has been hypothesized that the viral proteins (e.g., spike protein) infiltrate the neurons of the abducens nerve, causing neuroinflammation. Patients with COVID-19 who develop a sixth nerve palsy show atrophy of the lateral rectus muscle. Again, this is thought to be due to the neuroinflammatory nature of the virus, resulting in ocular myopathy.

The rare neurological complications documented in patients with COVID-19 are interesting to discuss, as they highlight the virus’s potential to affect the body in a multitude of ways. In the future, it would not be unreasonable to suggest that further neurological complications which have not yet been documented, will be observed.

## Conclusions

In conclusion, there are many different types of neurological complications that can occur in patients with COVID-19. The most common complications include anosmia, ageusia, and debilitating headaches. Whilst these are often short-lived, some studies have highlighted that there are patients who suffer from these problems chronically, which significantly impacts patient quality of life. Of the more worrying complications seen, cerebrovascular events are commonly seen. This includes both ischemic and hemorrhagic events within the arterial and/or venous vasculature. For ischemic events, COVID-19 causes a state of hypercoagulability, putting patients at a higher risk of developing TIA, stroke, or cerebral venous sinus thrombus. Ischemic events can also lead to hemorrhagic transformation. For purely hemorrhagic strokes, a combination of iatrogenic anticoagulation alongside the viral-induced inflammatory cascade puts patients at risk of developing a bleed. Therefore, both ischemic and hemorrhagic complications affect COVID-19 patients. The long-term outcomes following these events can be devastating. Some patients have better outcomes than others, but for those who experience widespread damage to the brain parenchyma, the outcomes are extremely poor, with high rates of morbidity and mortality. The neuroinflammatory complications of COVID-19 documented within the literature include meningitis, encephalitis, and encephalopathy. Whilst it is rare for these illnesses to be directly caused by SARS-CoV-2, the virus’s immunosuppressive nature means that individuals are susceptible to secondary neuroinflammatory complications, which can result in severe long-term brain damage. Furthermore, there is a fine balance between the neurological and psychiatric (i.e., neuropsychiatric) complications of COVID-19. Delirium is commonly observed and is precipitated by a range of factors including critical illness, ongoing infections, and environmental influences. The outcome for COVID patients who experience delirium has been documented to be worse than for those who do not. In rare cases, patients may develop a state of severe neuropsychiatric illness, for example, catatonia, although the exact pathological mechanisms for this are yet to be fully understood.

Looking at the peripheral neurological complications of COVID-19, critical illness neuropathy/myopathy has been observed, particularly in long-stay ICU patients. The impact this has on a patient’s wellbeing can be significant, as large amounts of time and effort are often spent to get the motor and sensory function of the limbs back to baseline. Like other viral infections, COVID-19 has shown the propensity to cause rare neurological complications. For example, Guillain-Barre, Miller Fischer syndrome, opsoclonus-myoclonus syndrome, and sixth nerve palsy. The significance of these complications is such that clinicians need to have these differentials in the back of their minds when patients present atypically. Overall, COVID-19 has been shown to cause an extensive range of neurological complications affecting both the CNS and PNS. Over time, scientific knowledge concerning the etiology, pathophysiology, and long-term outcomes of these complications will grow. As the virus continues to infect more individuals, novel neurological complications may be documented within the scientific literature. However, with the introduction of worldwide vaccination programs, the incidence of these complications will hopefully reduce over time, thus easing the burden on our ever-struggling healthcare system.
